# Supplementary material for: Dibenzofuran, 4-Chromanone, Acetophenone, and Dithiecine Derivatives: Cytotoxic Constituents from Eupatorium fortunei
Source: Int J Mol Sci. 2021 Jul 12;22(14):7448. doi: 10.3390/ijms22147448 (PMC8307038; doi:10.3390/ijms22147448)
Supplement: Supplementary file 1 [file ijms-22-07448-s001.zip › ijms-1255647-supplementary.pdf]

Supplementary Information

Dibenzofuran, 4-Chromanone, Acetophenone, and Dithiecine Derivatives: Cytotoxic Constituents from *Eupatorium fortunei*

Chun-Hao Chang [1], Semon Wu [2], Kai-Cheng Hsu [3,4], Wei-Jan Huang [5,6] and Jih-Jung Chen [7,8,9,*]

1 Institute of Biopharmaceutical Sciences, School of Pharmaceutical Sciences, National Yang Ming Chiao Tung University, Taipei 112, Taiwan; changch.ps08@nycu.edu.tw
2 Department of Life Science, Chinese Culture University, Taipei 110, Taiwan; wsm4@ulive.pccu.edu.tw
3 Graduate Institute of Cancer Biology and Drug Discovery, College of Medical Science and Technology, Taipei Medical University, Taipei 110, Taiwan; piki@tmu.edu.tw
4 Ph.D. Program for Cancer Molecular Biology and Drug Discovery, College of Medical Science and Technology, Taipei Medical University, Taipei 110, Taiwan
5 Ph.D. Program in Biotechnology Research and Development, College of Pharmacy, Taipei Medical University, Taipei 110, Taiwan; wjhuang@tmu.edu.tw
6 Graduate Institute of Pharmacognosy, College of Pharmacy, Taipei Medical University, Taipei 110, Taiwan
7 Department of Pharmacy, School of Pharmaceutical Sciences, National Yang Ming Chiao Tung University, Taipei 112, Taiwan
8 Faculty of Pharmacy, National Yang-Ming University, Taipei 112, Taiwan
9 Department of Medical Research, China Medical University Hospital, China Medical University, Taichung 404, Taiwan
* Correspondence: jjungchen@nycu.edu.tw; Tel: +886-2-2826-7195; Fax: +886-2-2823-2940

Table S1. Calculated physicochemical properties of compound **1**.

Property	Value
Number of H-bond acceptors	4
Number of H-bond donors	2
Molecular weight	346.38 g/mol
ALogP98	5.08
Molecular surface area	347.01 Å^2
Molecular polar surface area	70.67 Å^2
ADMET absorption level	Good

Table S2. Predicted targets of compound **1**.

Target	Description	Max Tc	P-Value
PON1	Serum paraoxonase/arylesterase 1	0.34	2.784e-21
CELA1	Chymotrypsin-like elastase family member 1	0.41	1.144e-14
CBR1	Carbonyl reductase [NADPH] 1	0.31	3.485e-08
NQO1	NAD(P)H dehydrogenase [quinone] 1	0.31	7.109e-08

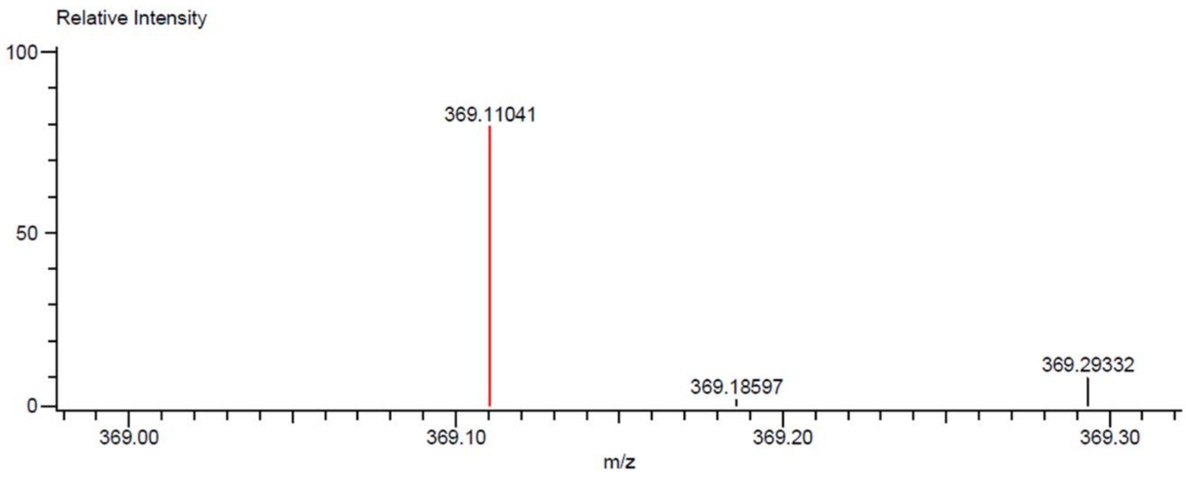

Mass	Intensity	Calc. Mass	Mass Difference [mDa]	Mass Difference [ppm]	Possible Formula
369.11041	2214.69	369.11028	0.13	0.35	$^{12}C_{22}{}^{1}H_{18}{}^{23}Na_{1}{}^{16}O_{4}$

Figure S1. HR-ESI-MS spectrum of **1**.

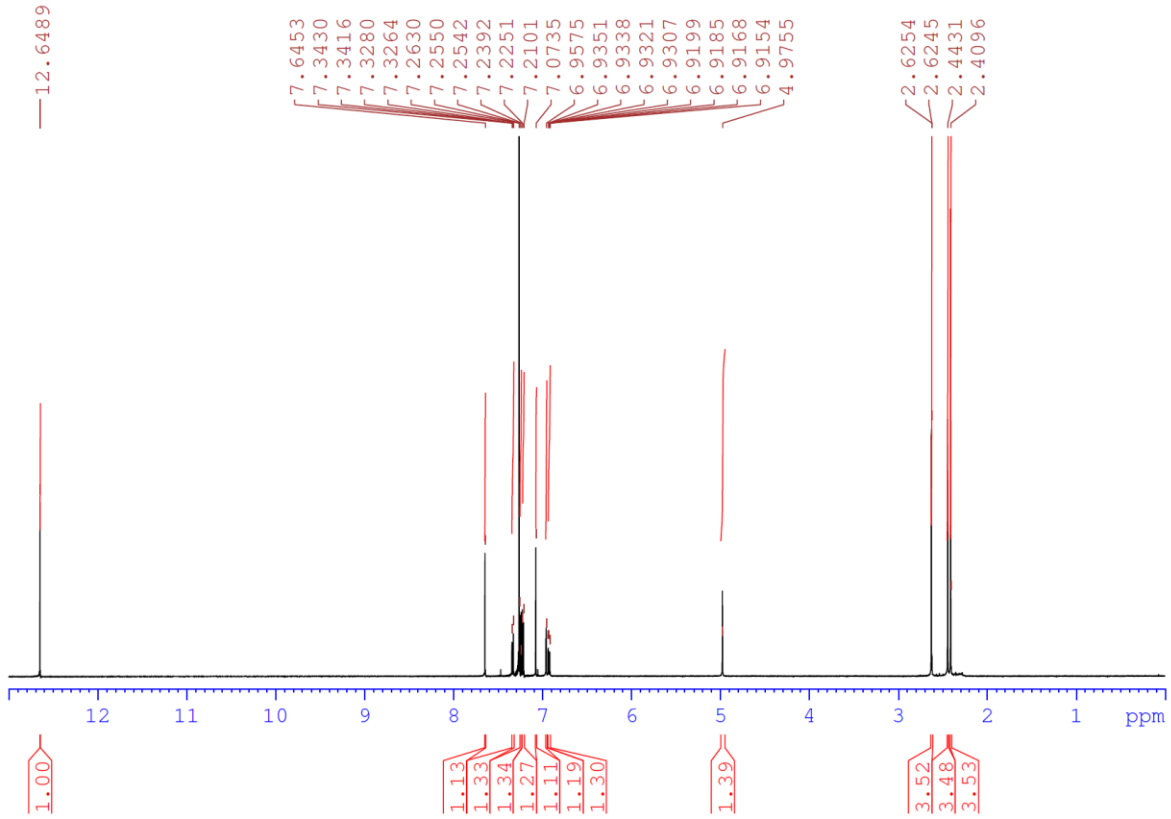

Figure S2. ^{1}H-NMR spectrum (CDCl3, 500 MHz) of **1**.

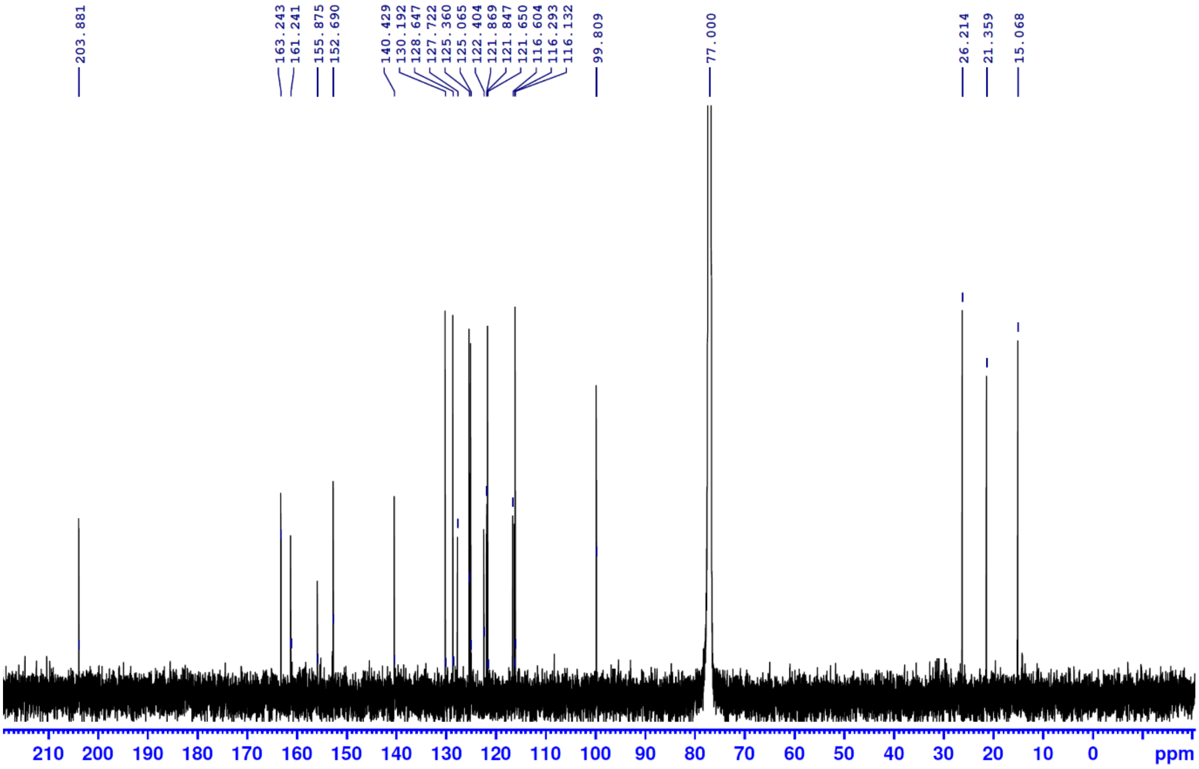

Figure S3. ^{13}C-NMR spectrum (CDCl3, 125 MHz) of **1.**

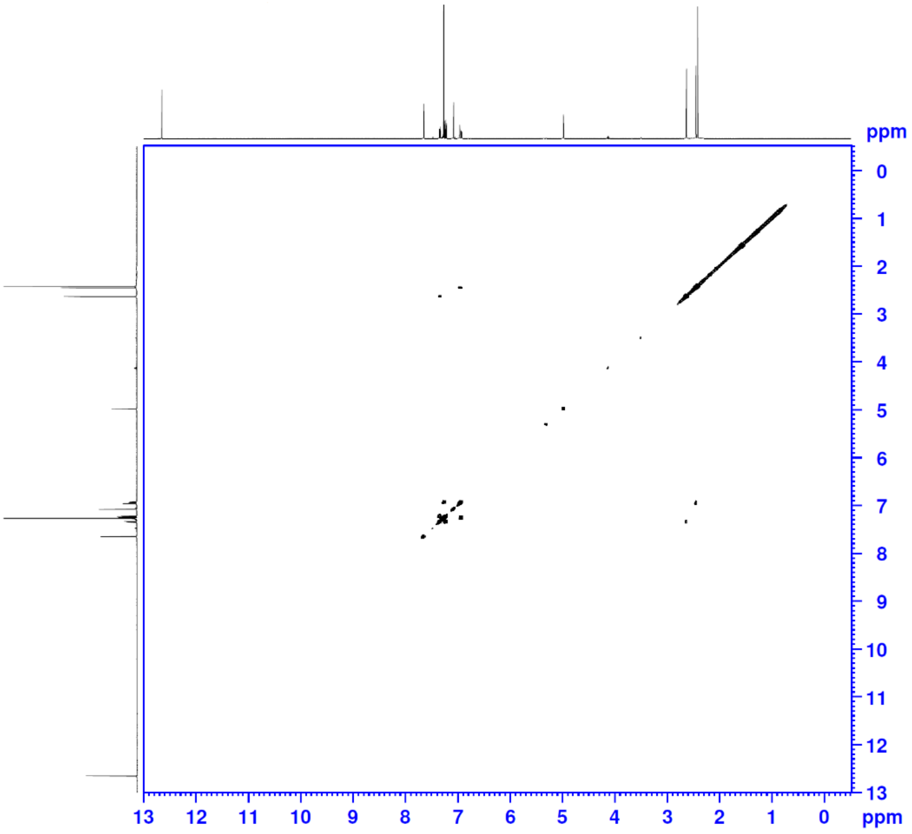

Figure S4. ^{1}H–^{1}H COSY spectrum of **1**.

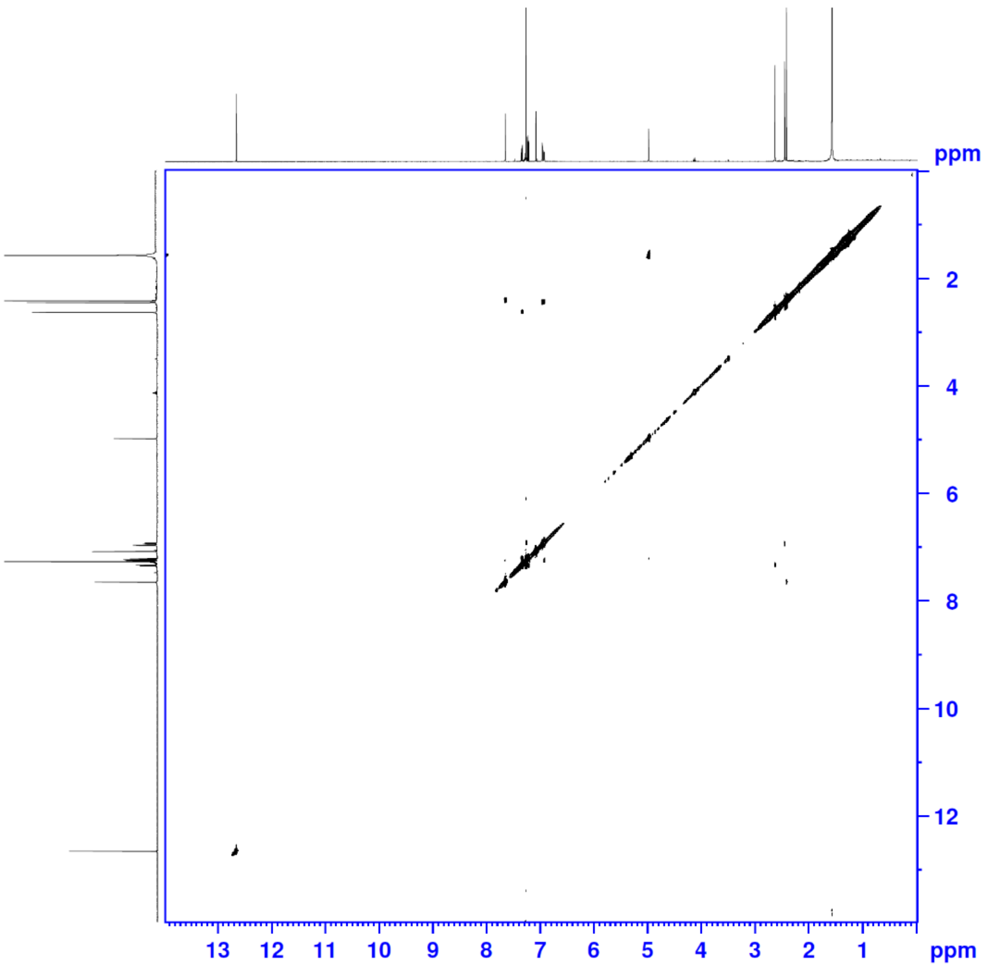

Figure S5. ROESY spectrum of **1**.

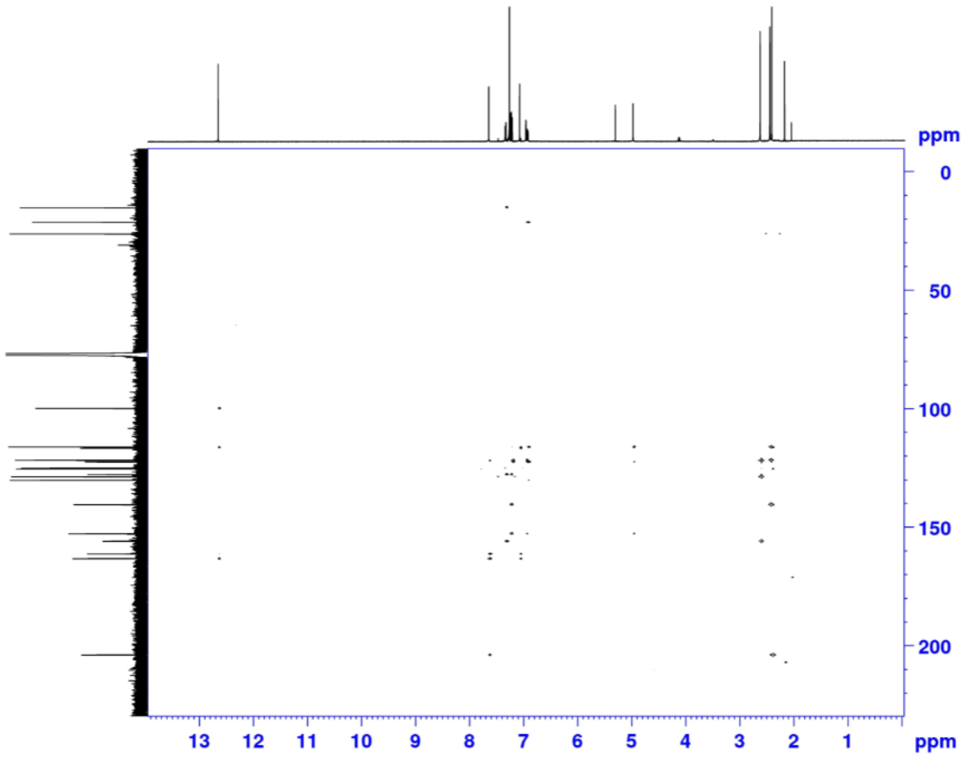

Figure S6. HMBC spectrum of **1**.

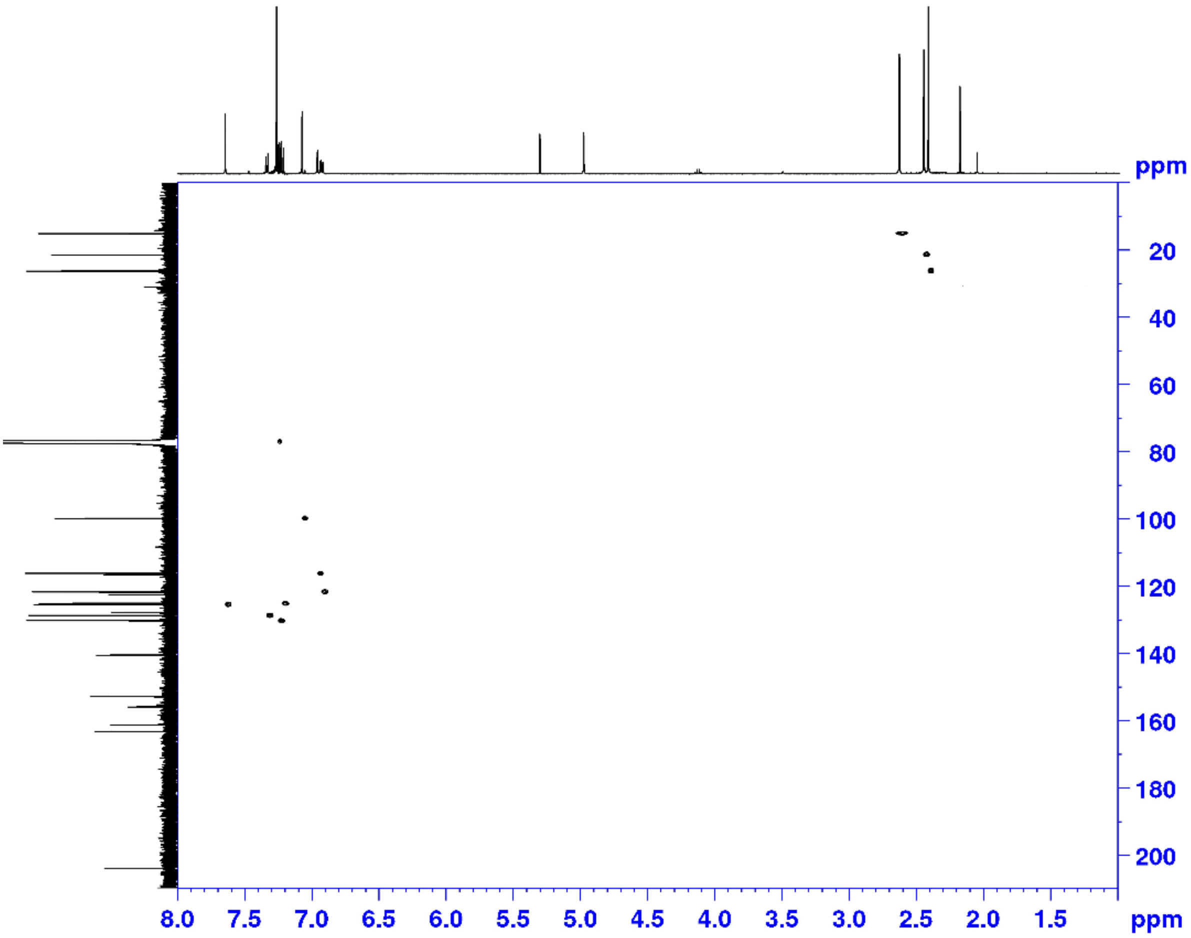

Figure S7. HSQC spectrum of **1**.

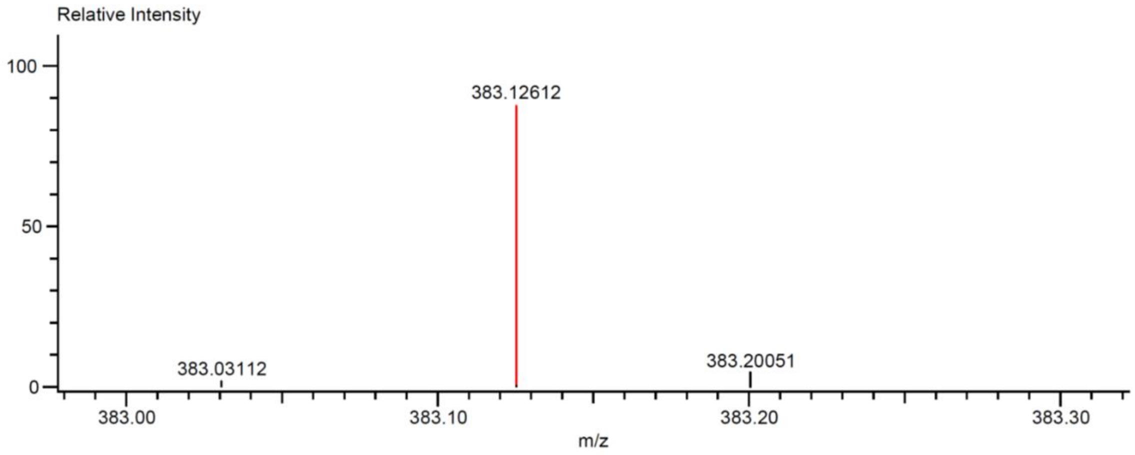

Mass	Intensity	Calc. Mass	Mass Difference [mDa]	Mass Difference [ppm]	Possible Formula
383.12612	2282.32	383.12593	0.19	0.49	$^{12}C_{23}{}^{1}H_{20}{}^{23}Na_{1}{}^{16}O_{4}$

Figure S8. HR-ESI-MS spectrum of **2**.

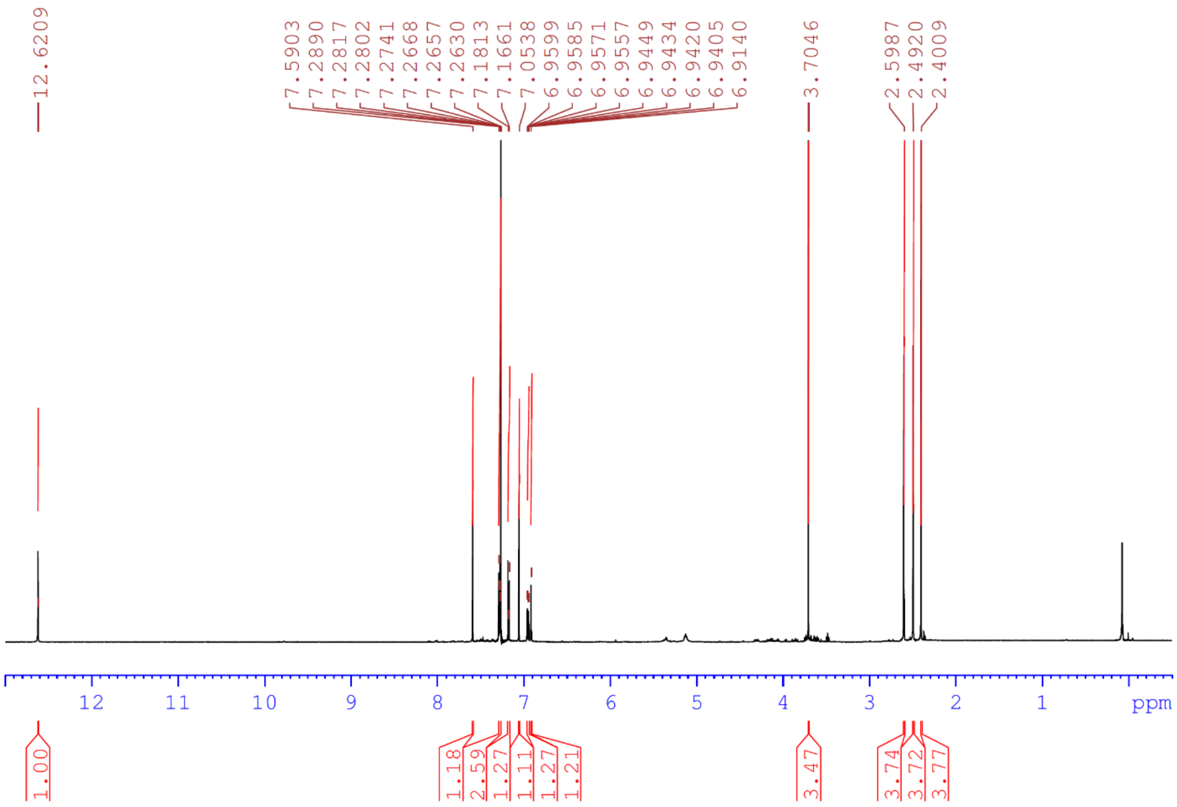

Figure S9. ^{1}H-NMR spectrum ($CDCl_3$, 500 MHz) of **2**.

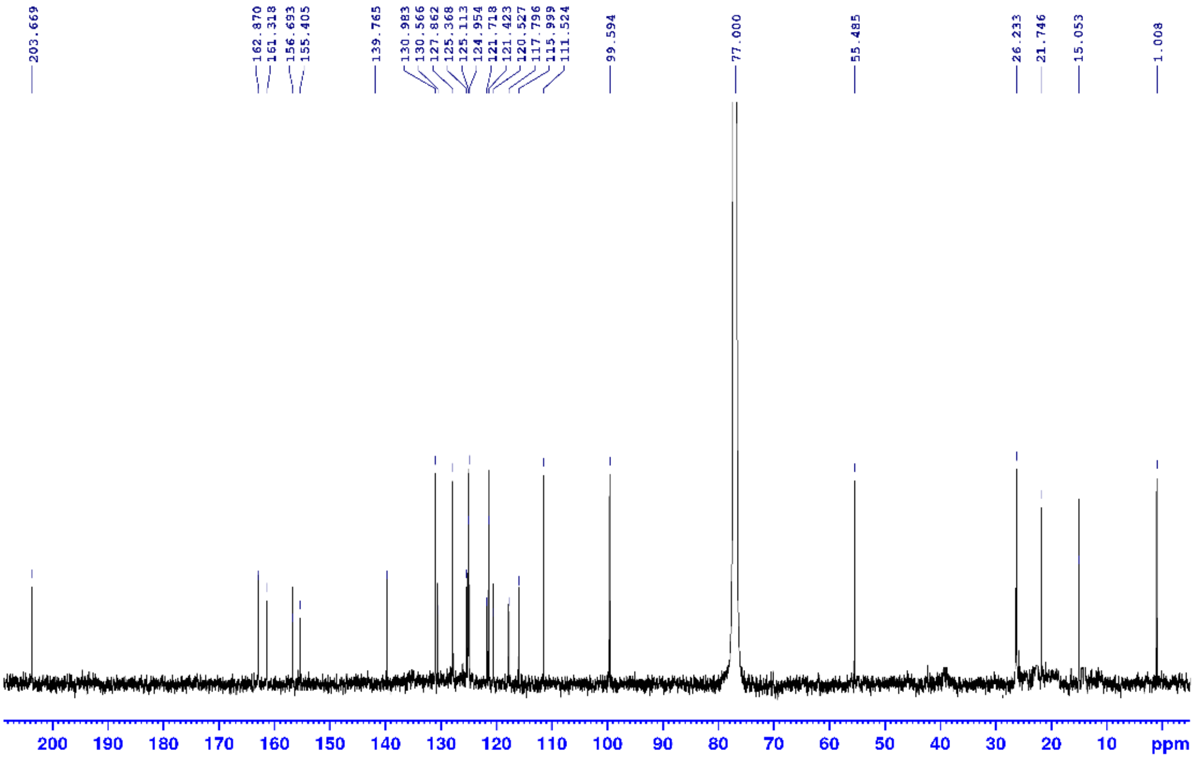

Figure S10. ^{13}C-NMR spectrum ($CDCl_3$, 125 MHz) of **2**.

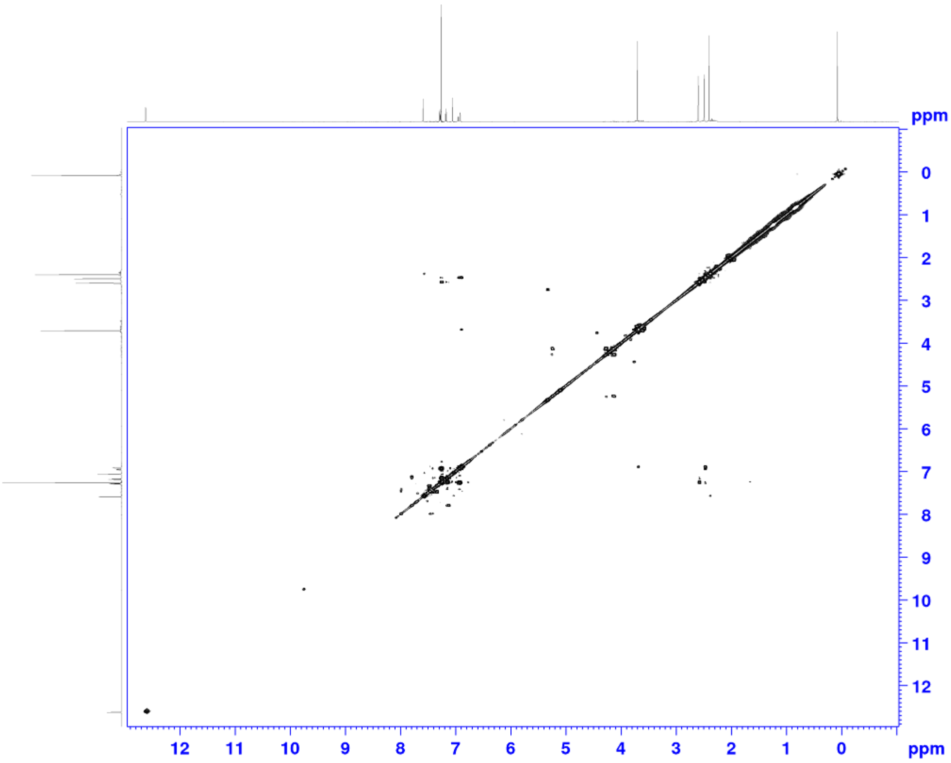

Figure S11. ^1H–^1H COSY spectrum of **2**.

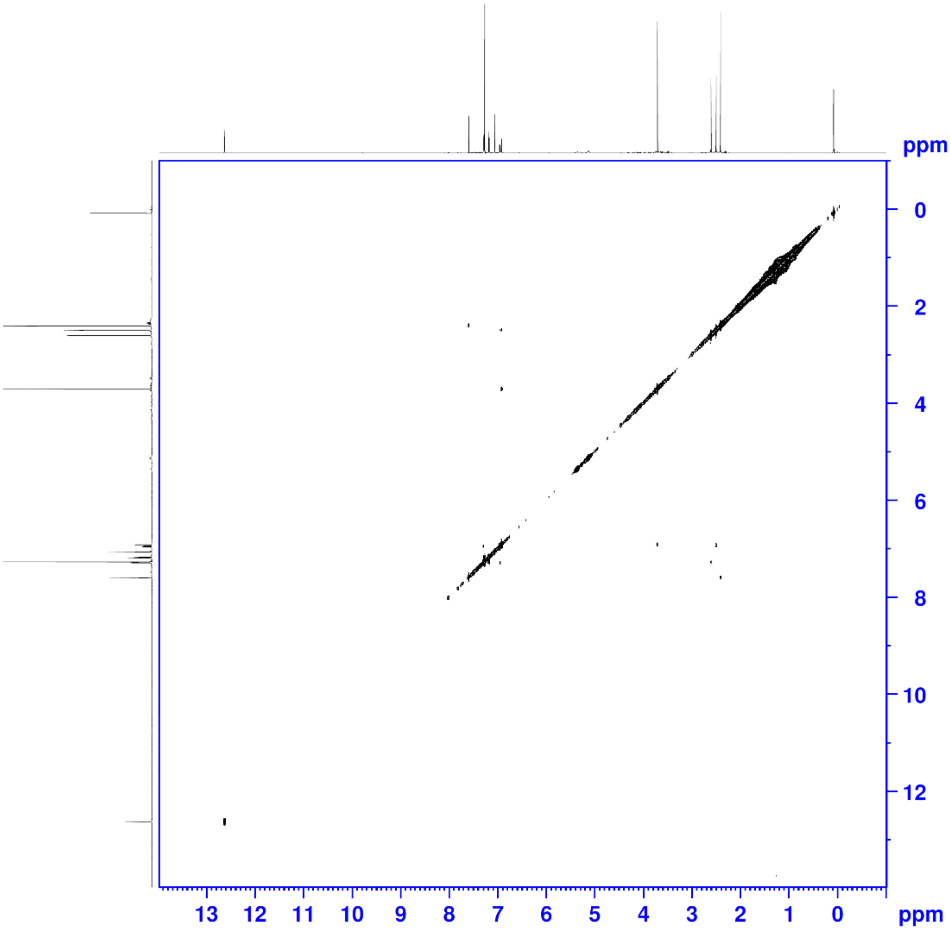

Figure S12. ROESY spectrum of **2**.

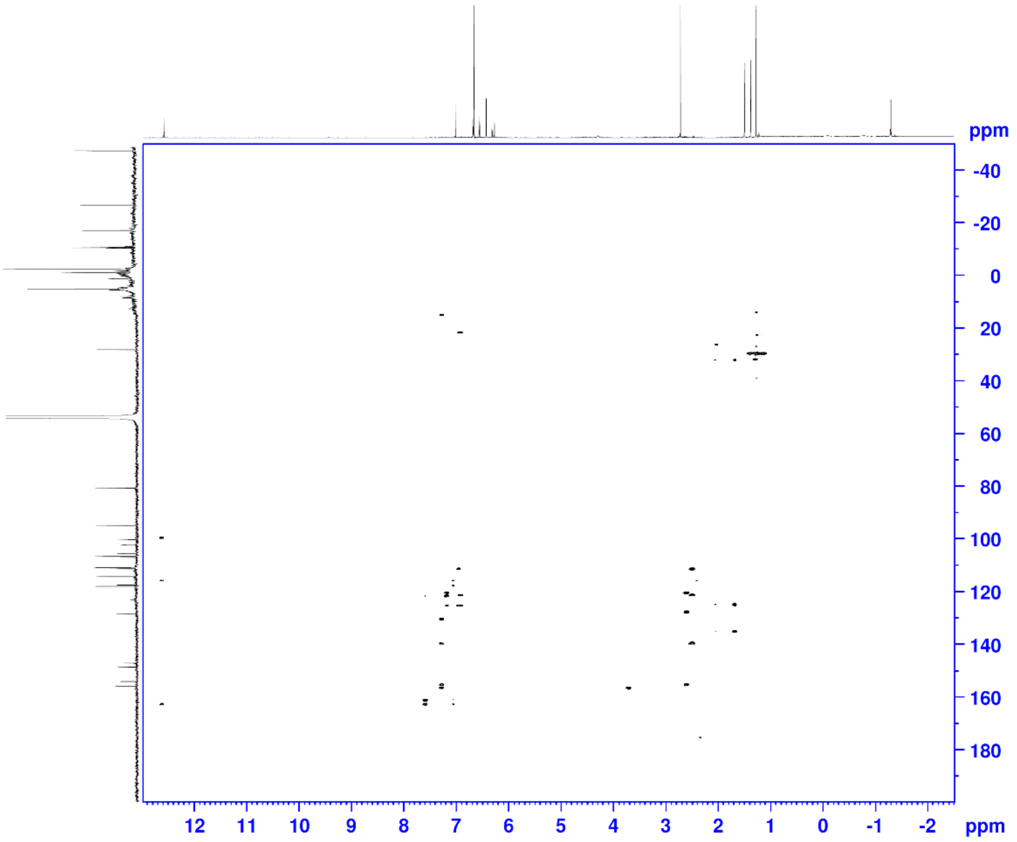

Figure S13. HMBC spectrum of **2**.

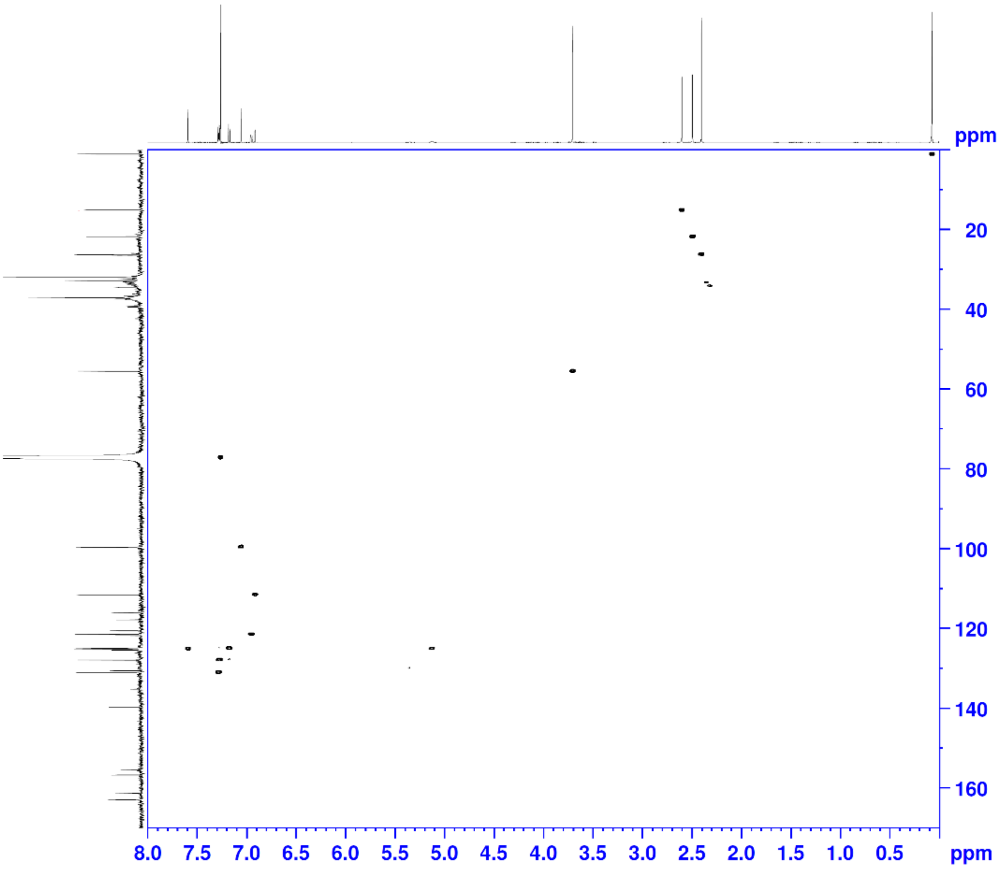

Figure S14. HSQC spectrum of **2**.

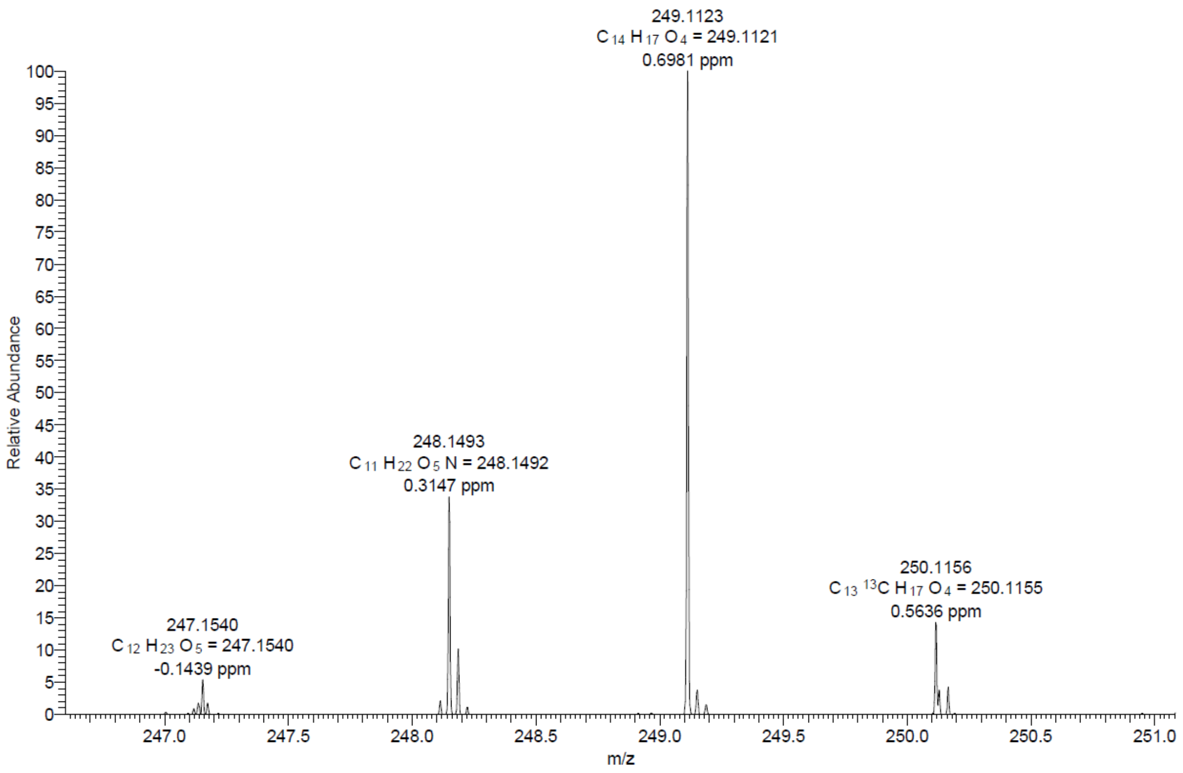

Figure S15. HR-ESI-MS spectrum of **3**.

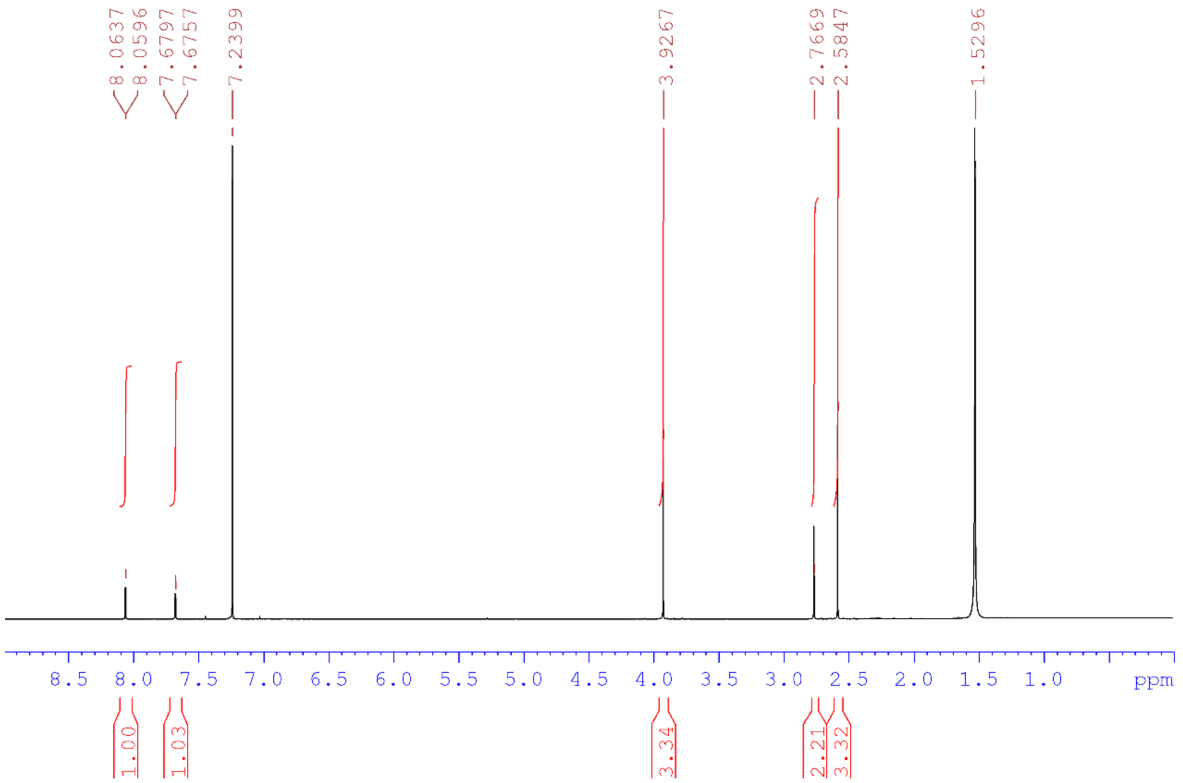

Figure S16. ^{1}H-NMR spectrum ($CDCl_3$, 500 MHz) of **3**.

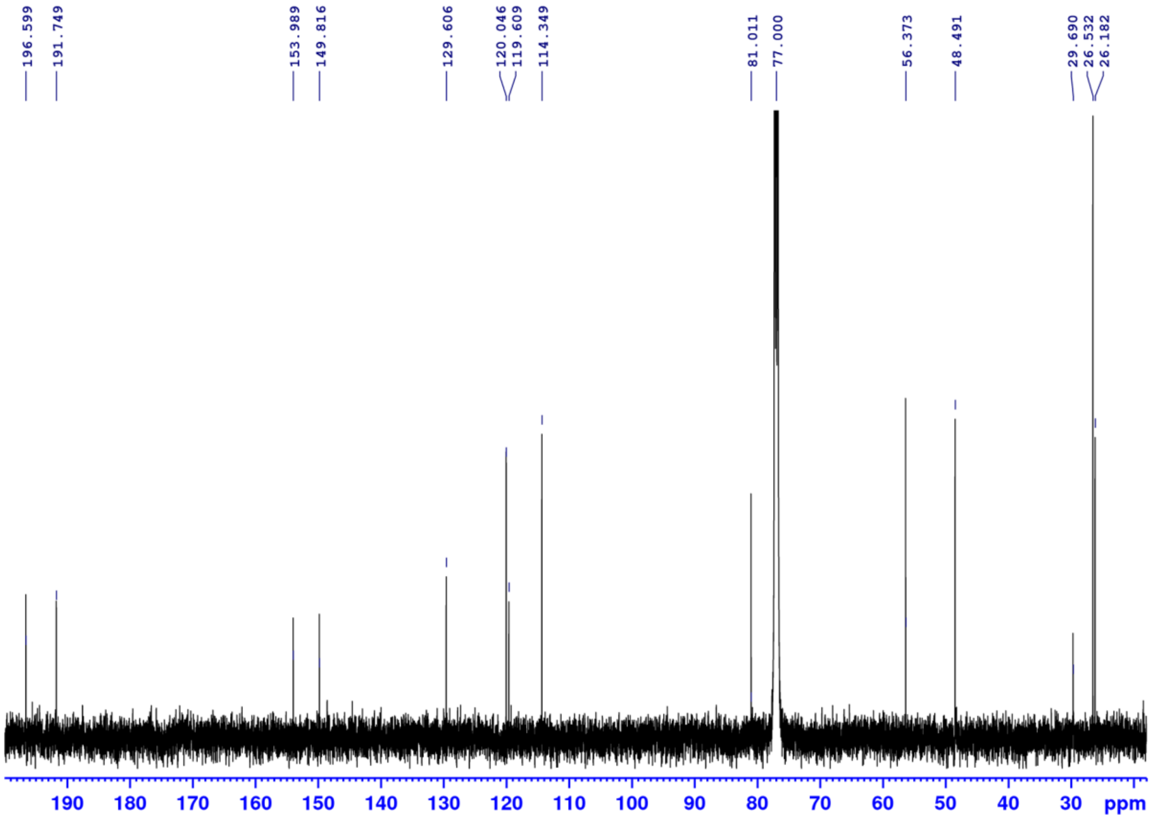

Figure S17. ^{13}C-NMR spectrum ($CDCl_3$, 125 MHz) of **3**.

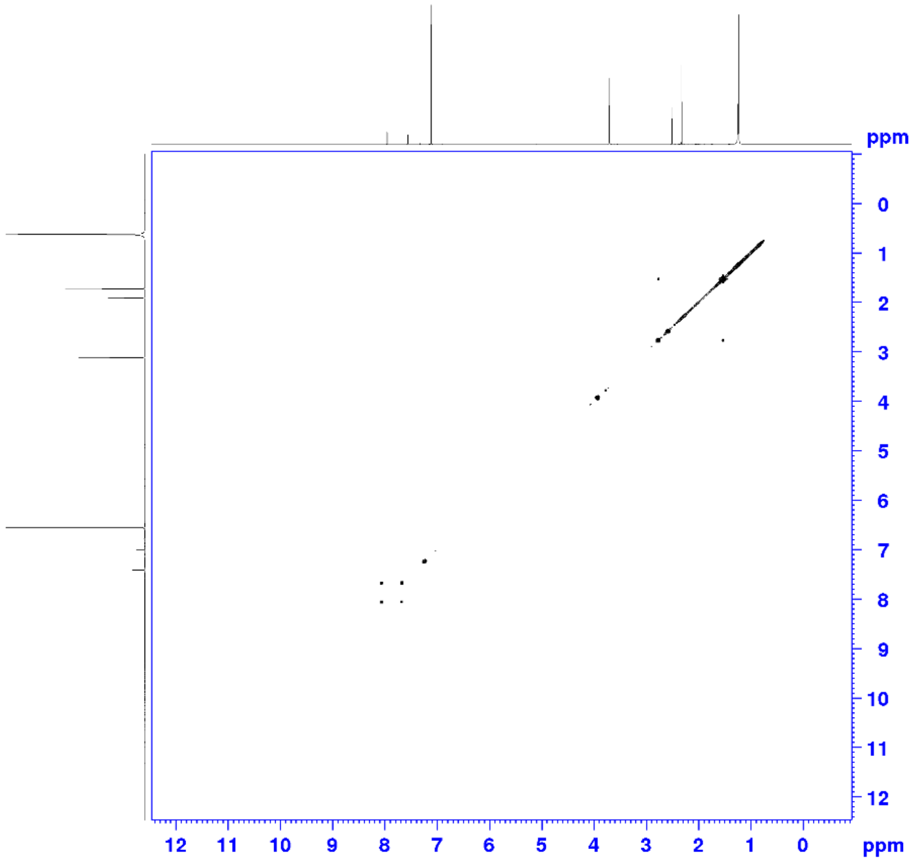

Figure S18. ^{1}H–^{1}H COSY spectrum of **3**.

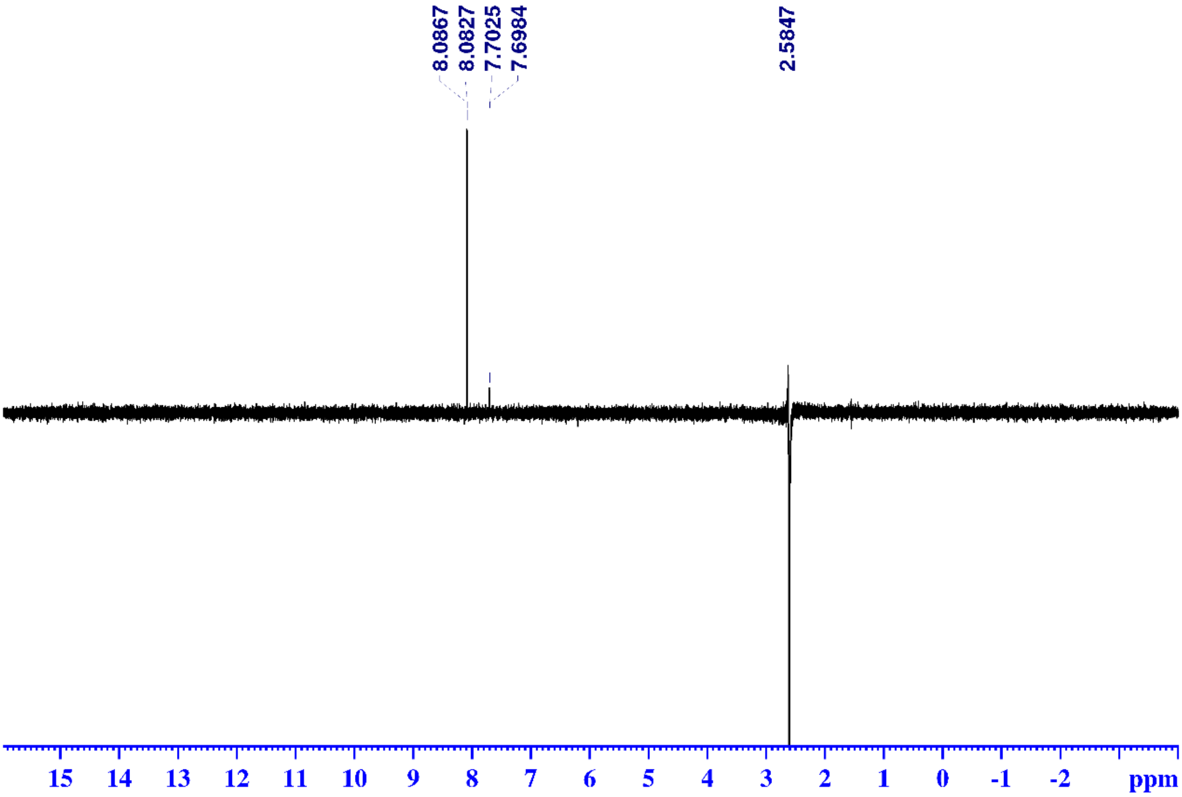

Figure S19. 1D selective NOESY (δ_H 2.58) spectrum of **3**.

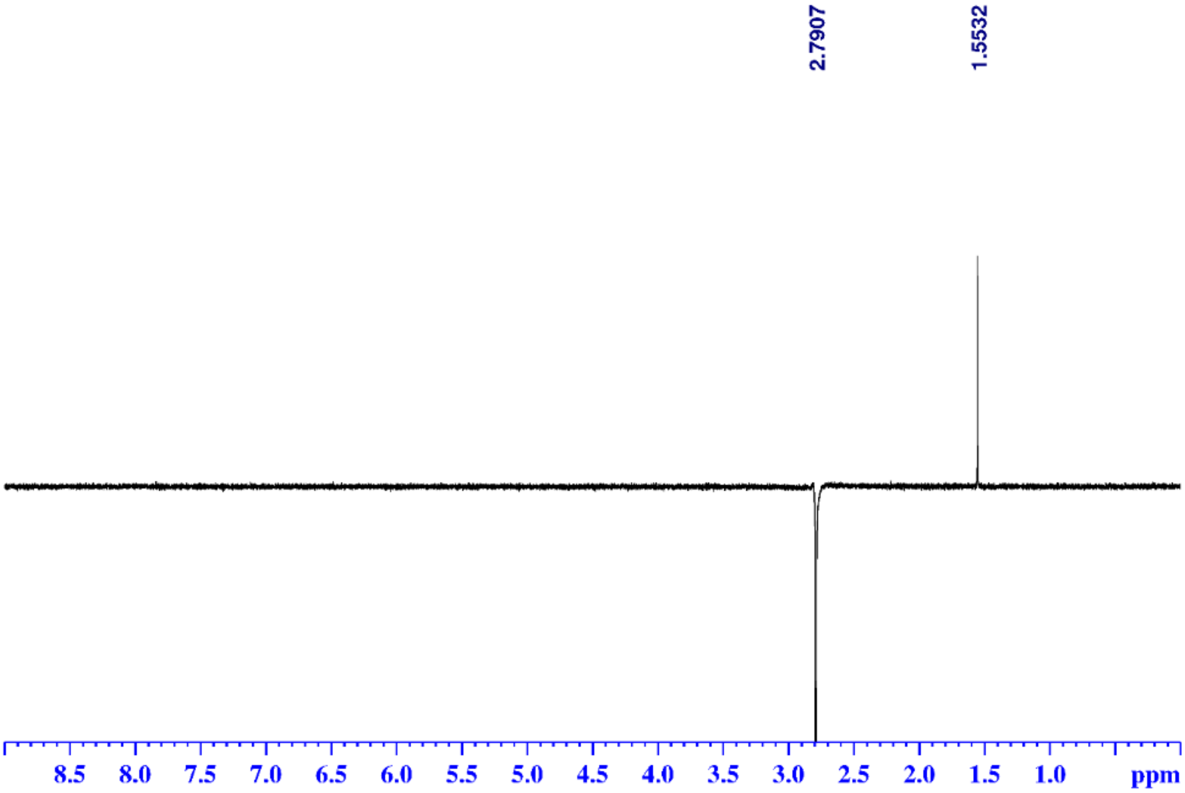

Figure S20. 1D selective NOESY (δ_H 2.79) spectrum of **3**.

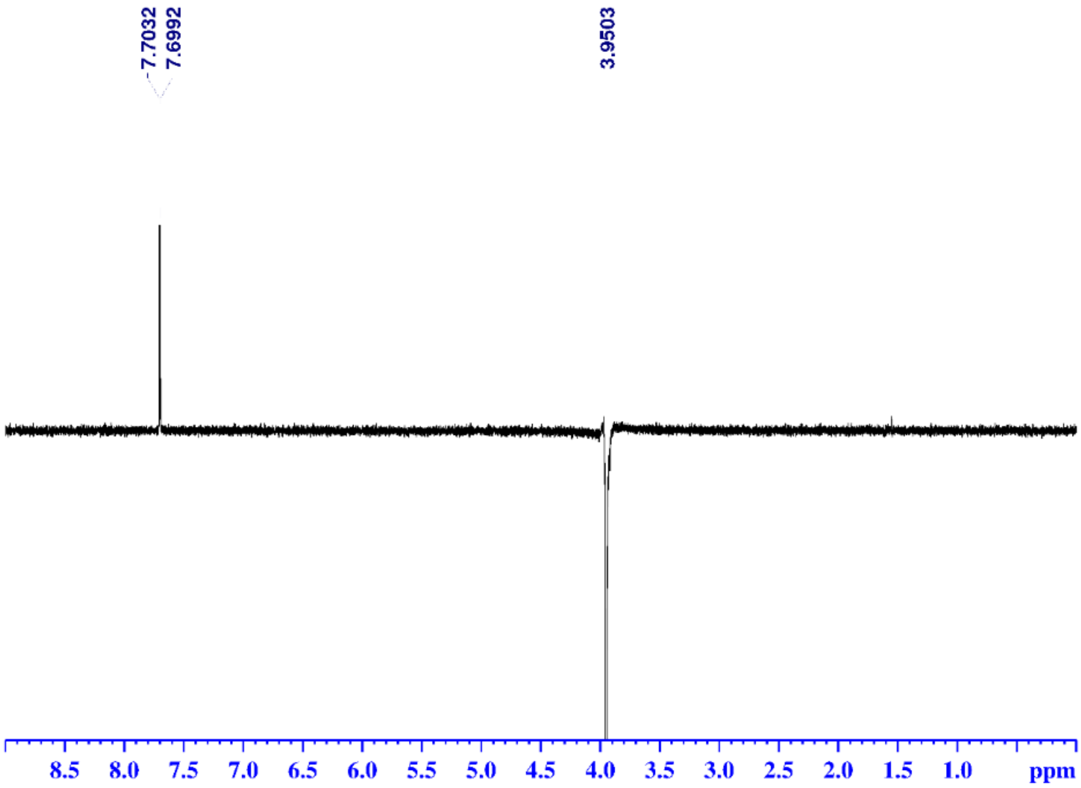

Figure S21. 1D selective NOESY (δ_H 3.95) spectrum of **3**.

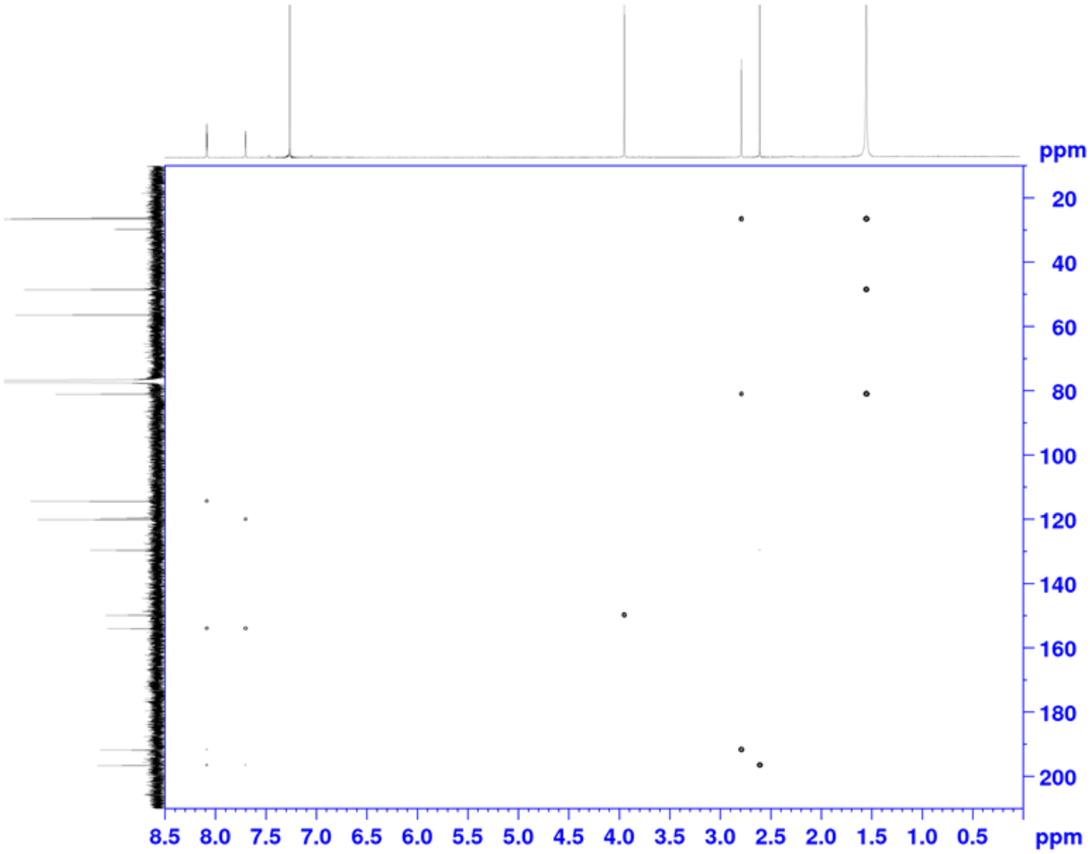

Figure S22. HMBC spectrum of **3**.

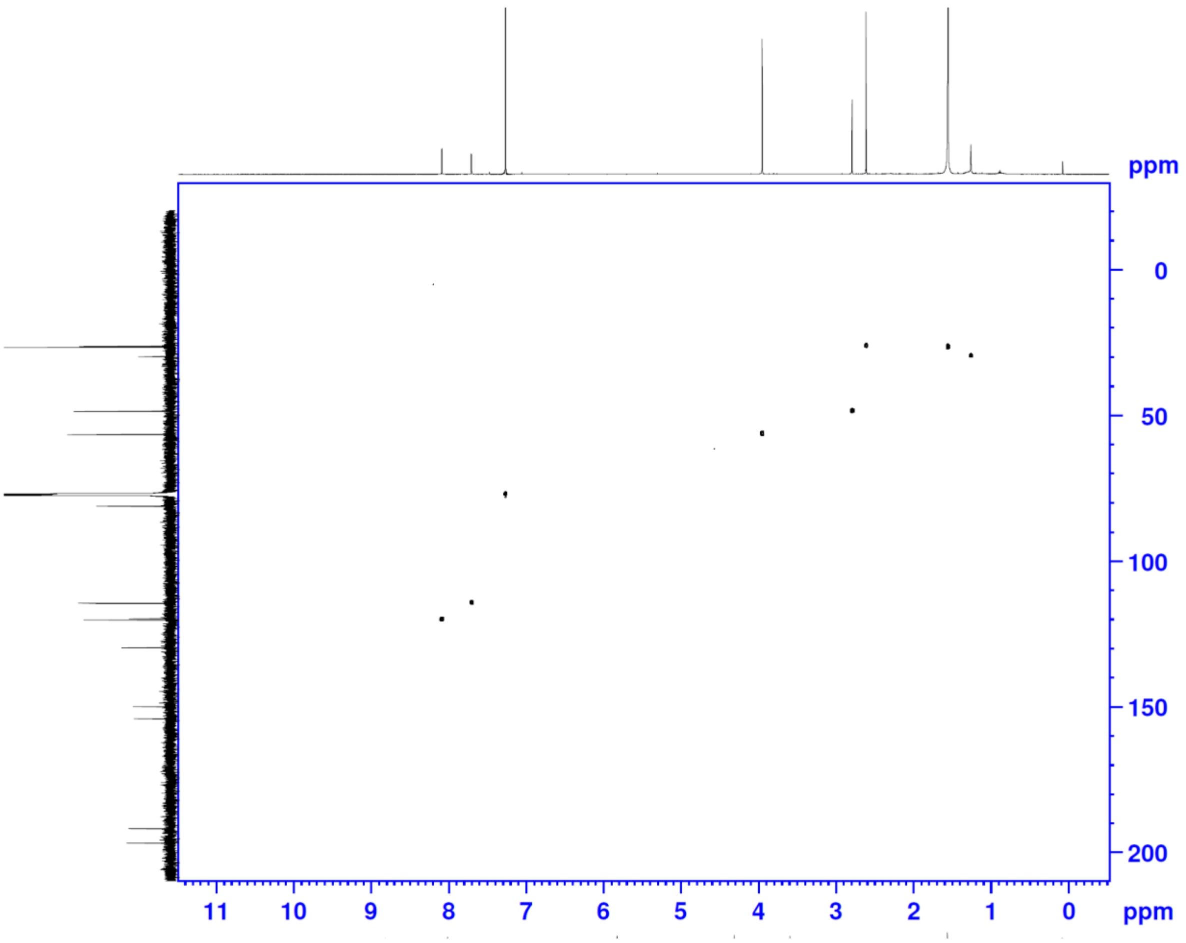

Figure S23. HSQC spectrum of **3**.

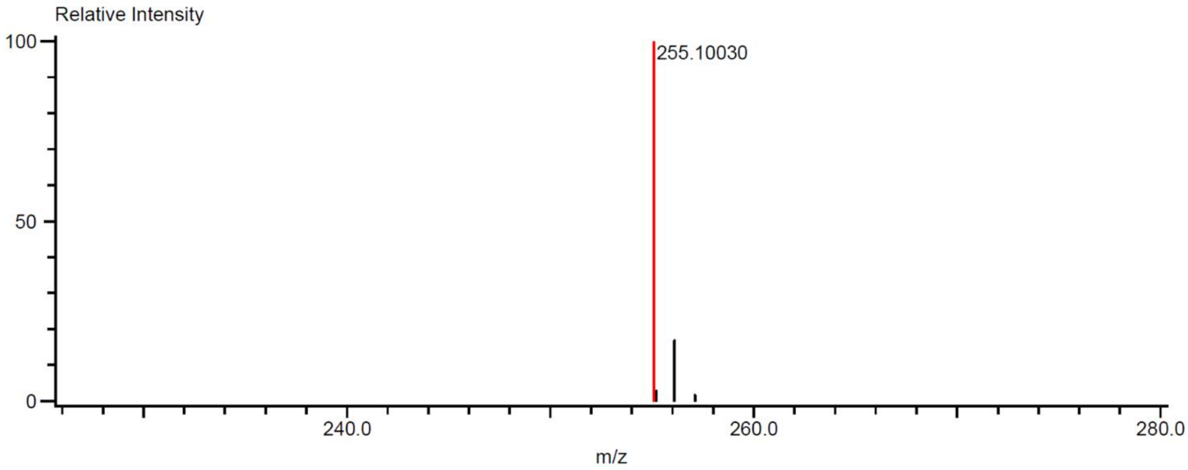

Mass	Intensity	Calc. Mass	Mass Difference [mDa]	Mass Difference [ppm]	Possible Formula
255.10030	30162.90	255.09971	0.58	2.29	$^{12}C_{14}{}^{1}H_{16}{}^{23}Na_{1}{}^{16}O_{3}$

Figure S24. HR-ESI-MS spectrum of **4**.

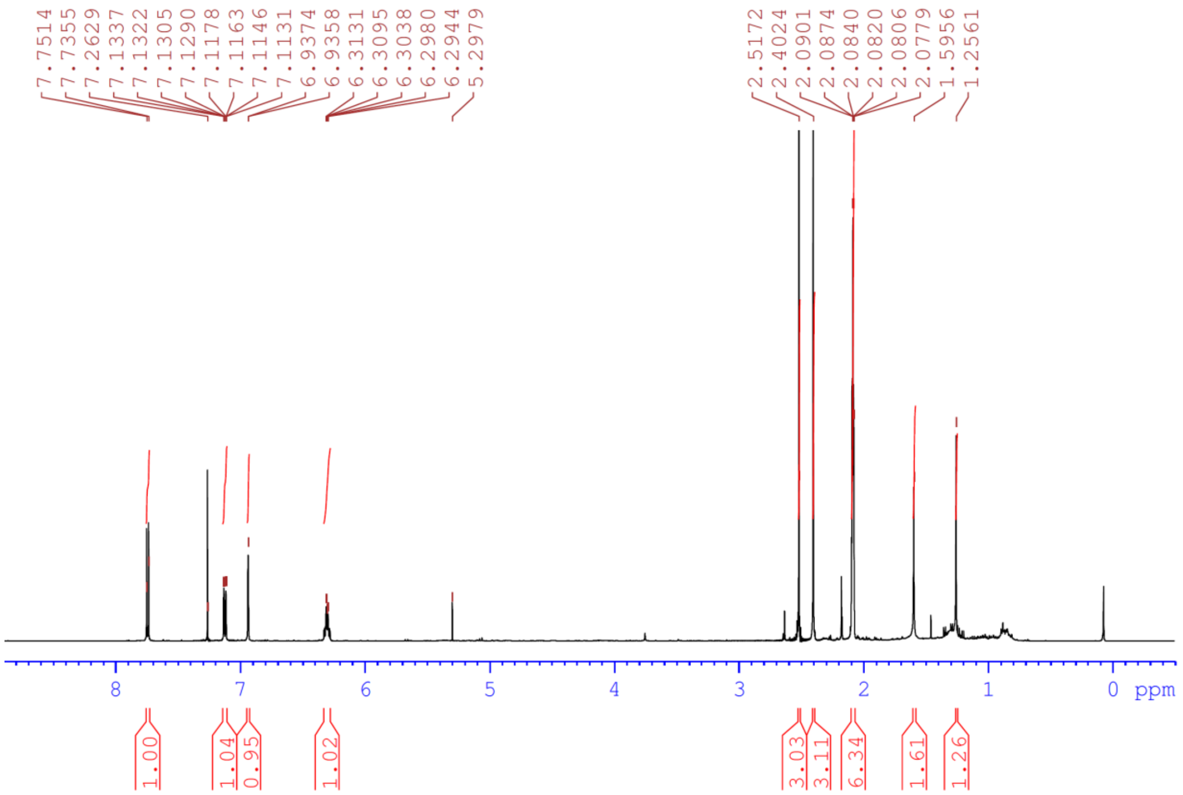

Figure S25. ^{1}H-NMR spectrum ($CDCl_3$, 500 MHz) of **4**.

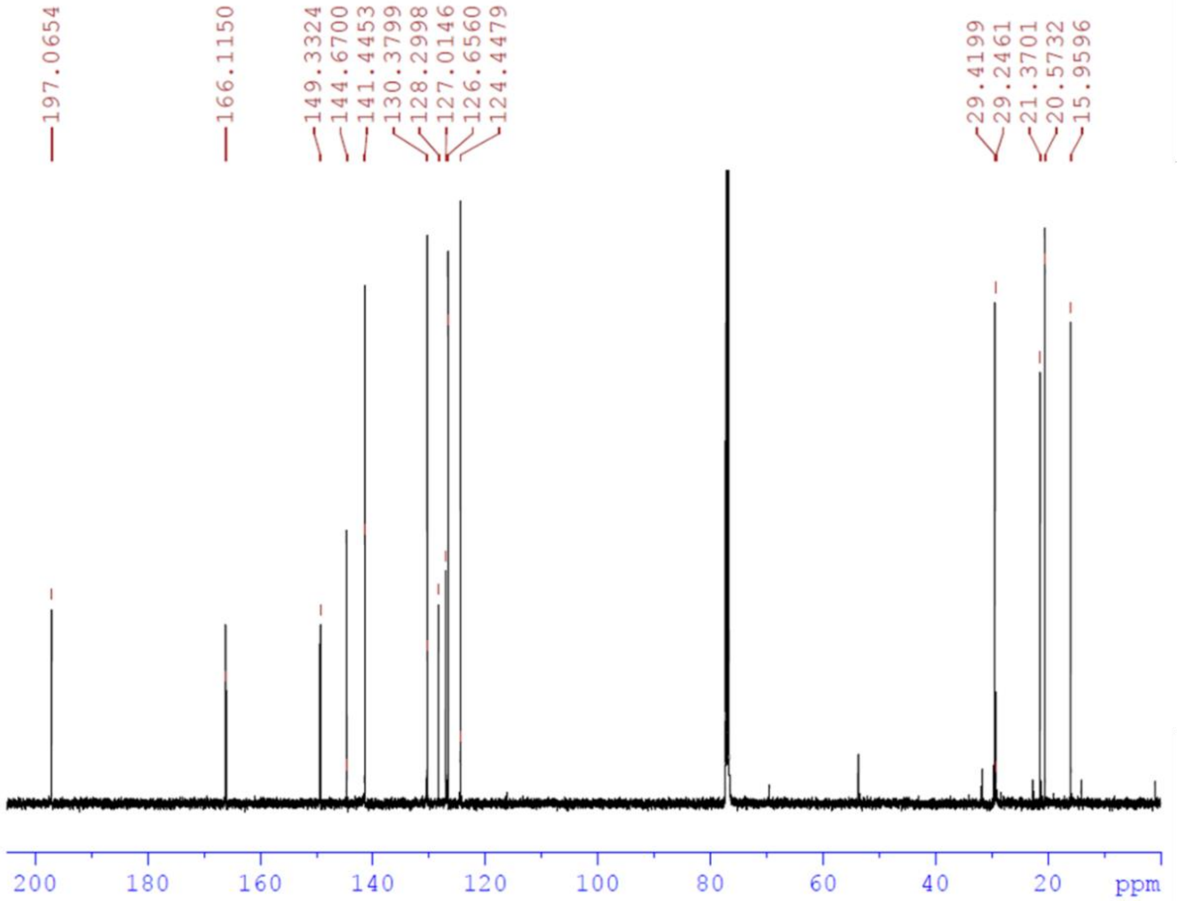

Figure S26. ^{13}C-NMR ($CDCl_3$, 125 MHz) spectrum of **4**.

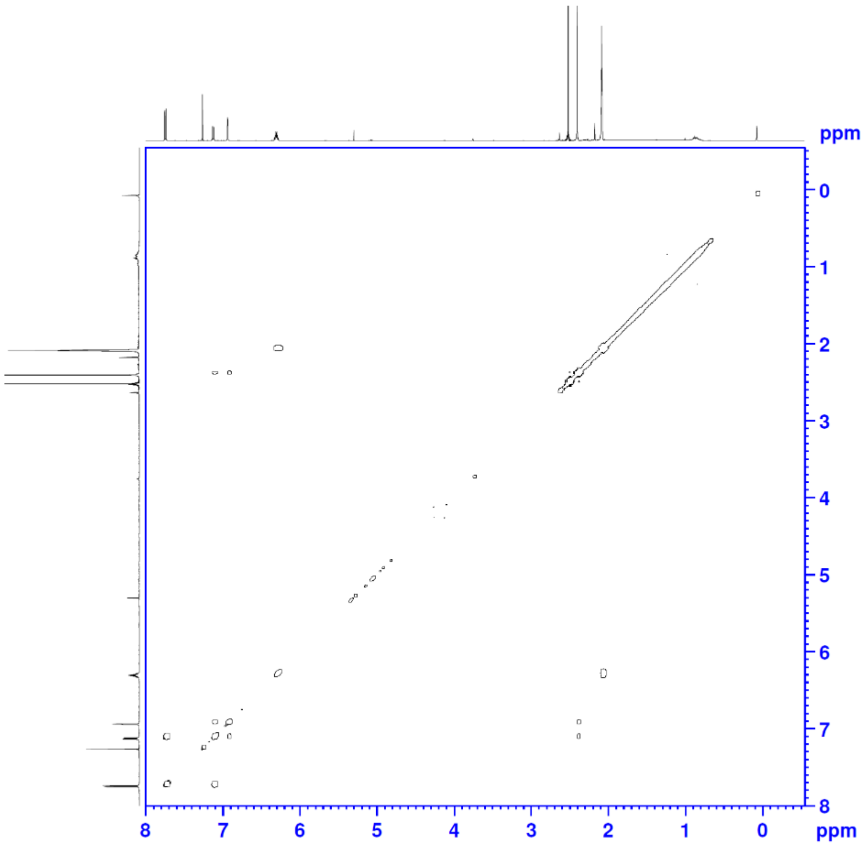

Figure S27. ^{1}H–^{1}H COSY spectrum of **4**.

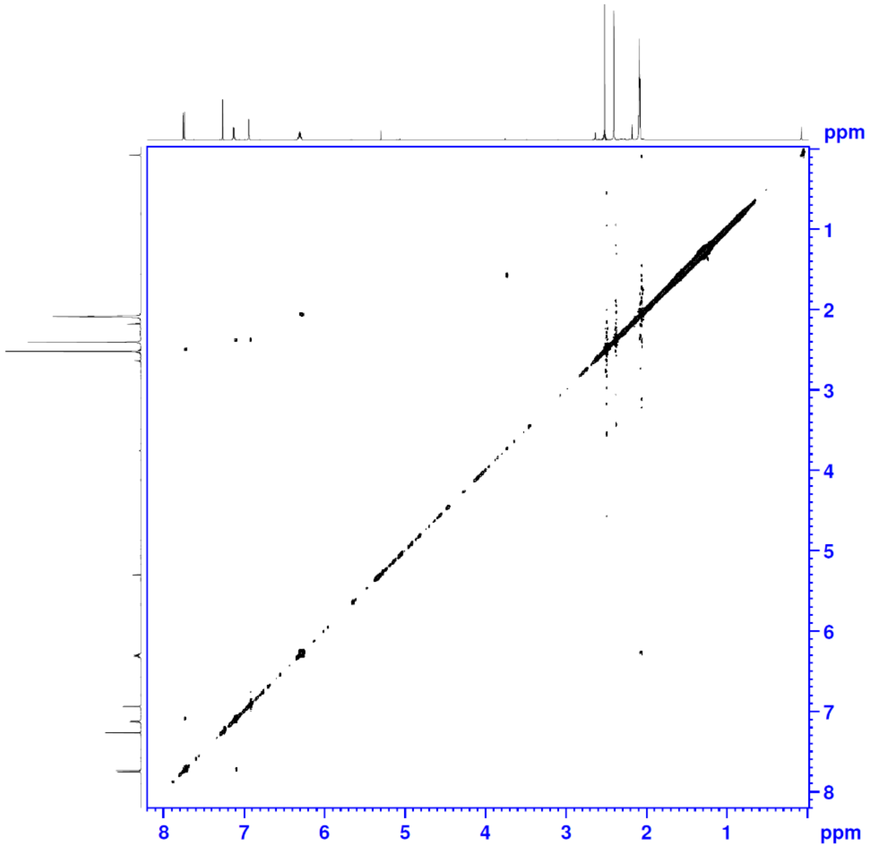

Figure S28. ROESY spectrum of **4**.

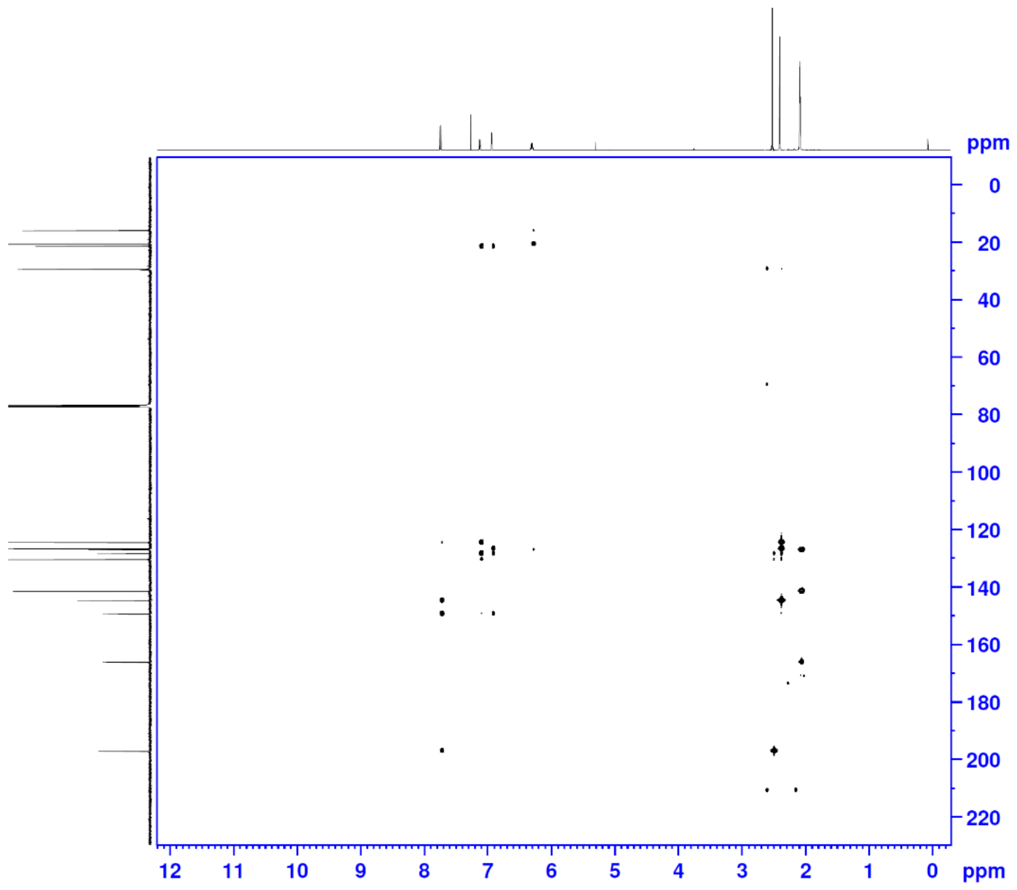

Figure S29. HMBC spectrum of **4**.

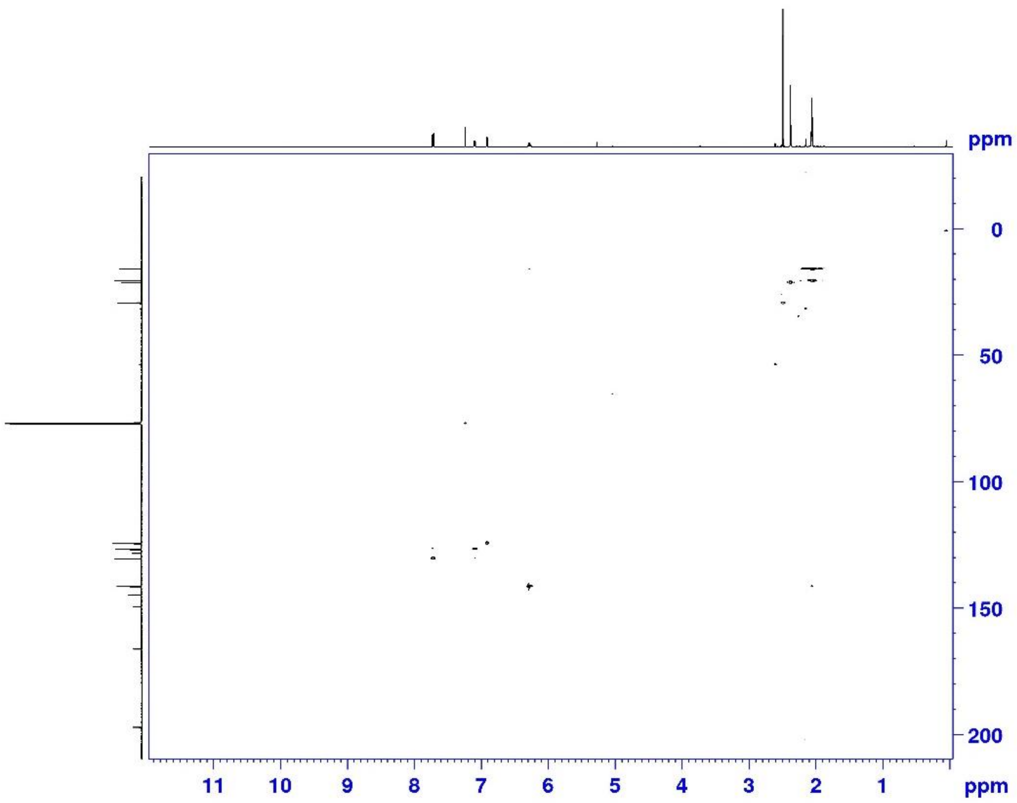

Figure S30. HSQC spectrum of **4**.

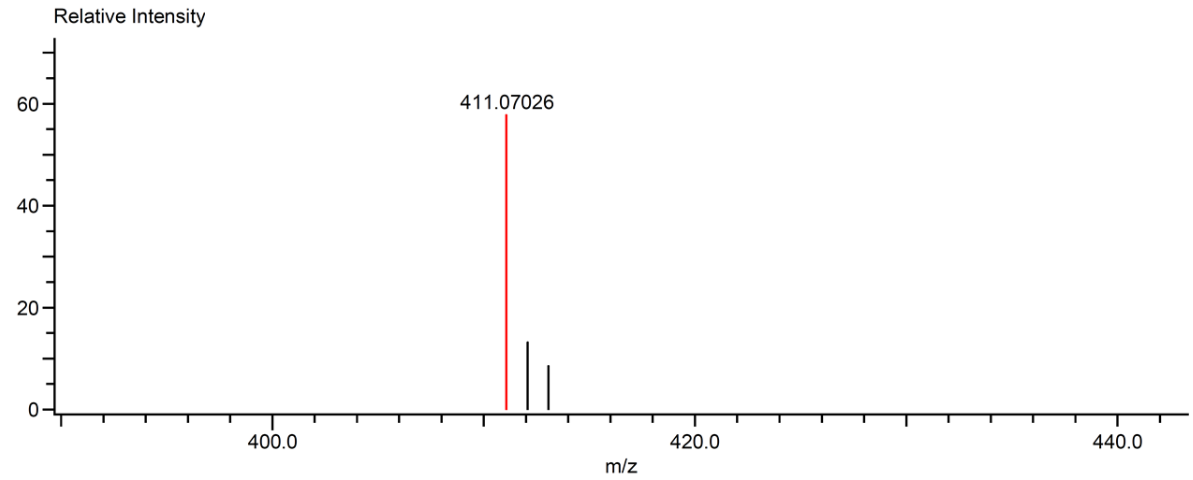

Mass	Intensity	Calc. Mass	Mass Difference [mDa]	Mass Difference [ppm]	Possible Formula
411.07026	7792.70	411.07007	0.20	0.47	$^{12}C_{20}{}^{1}H_{20}{}^{23}Na_{1}{}^{16}O_{4}{}^{32}S_{2}$

Figure S31. HR-ESI-MS spectrum of **5**.

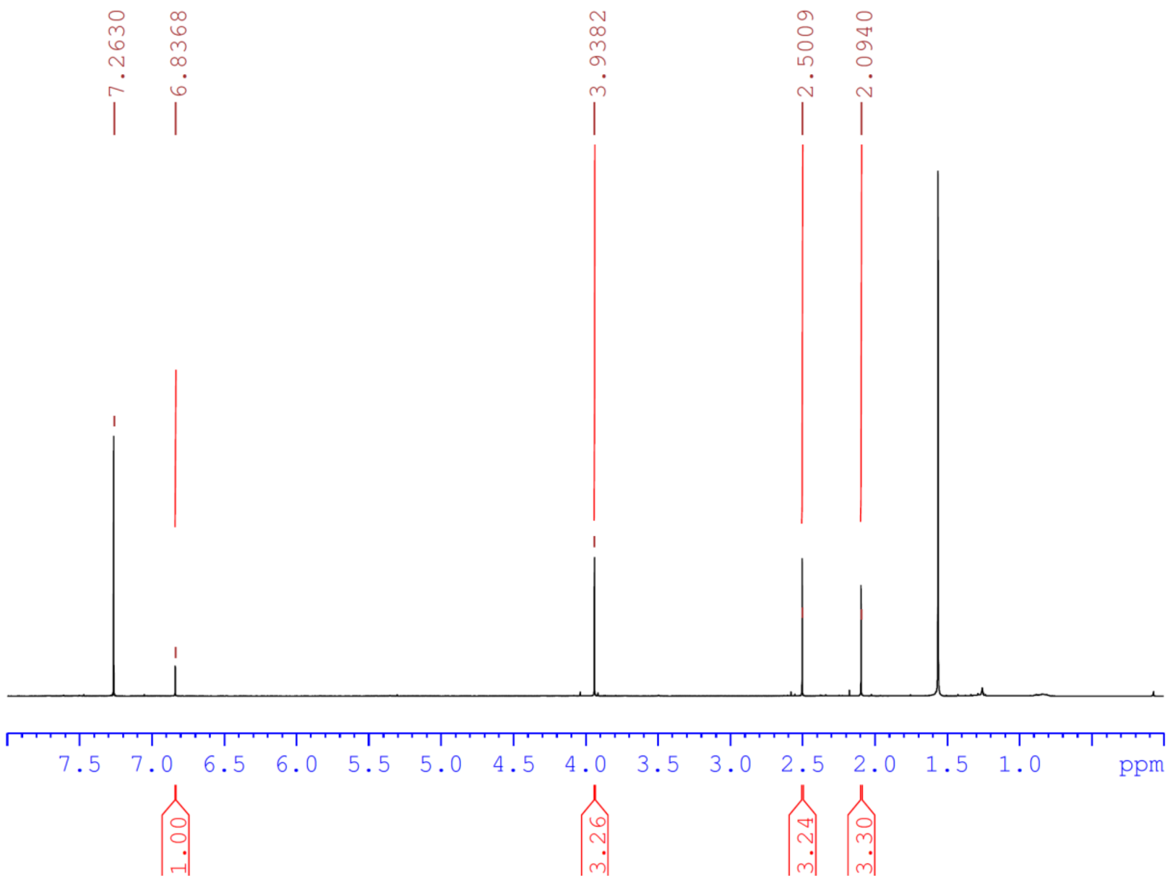

Figure S32. ^{1}H-NMR spectrum ($CDCl_3$, 500 MHz) of **5**.

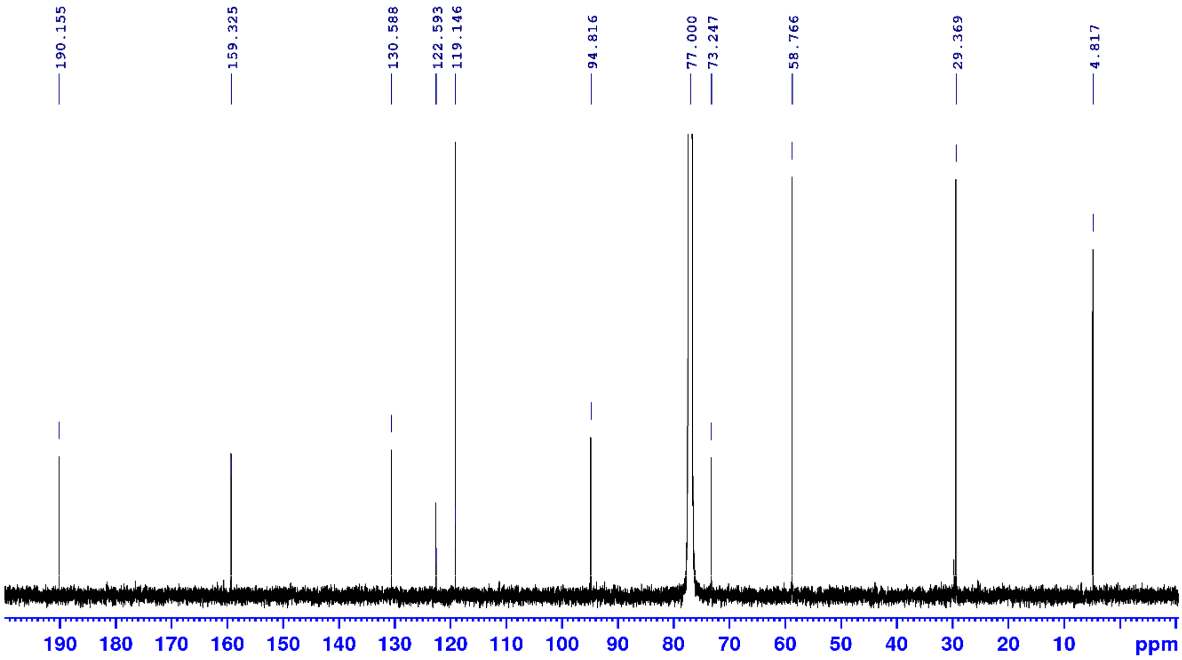

Figure S33. ^{13}C-NMR spectrum ($CDCl_3$, 125 MHz) of **5**.

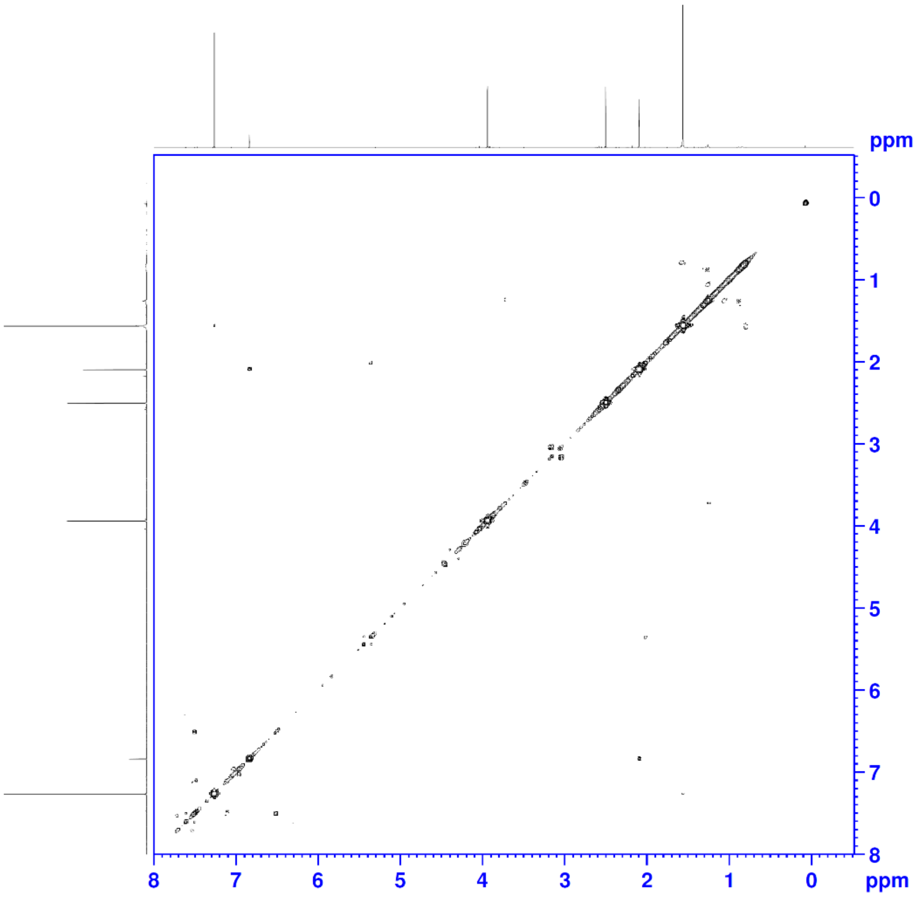

Figure S34. ^{1}H–^{1}H COSY spectrum of **5**.

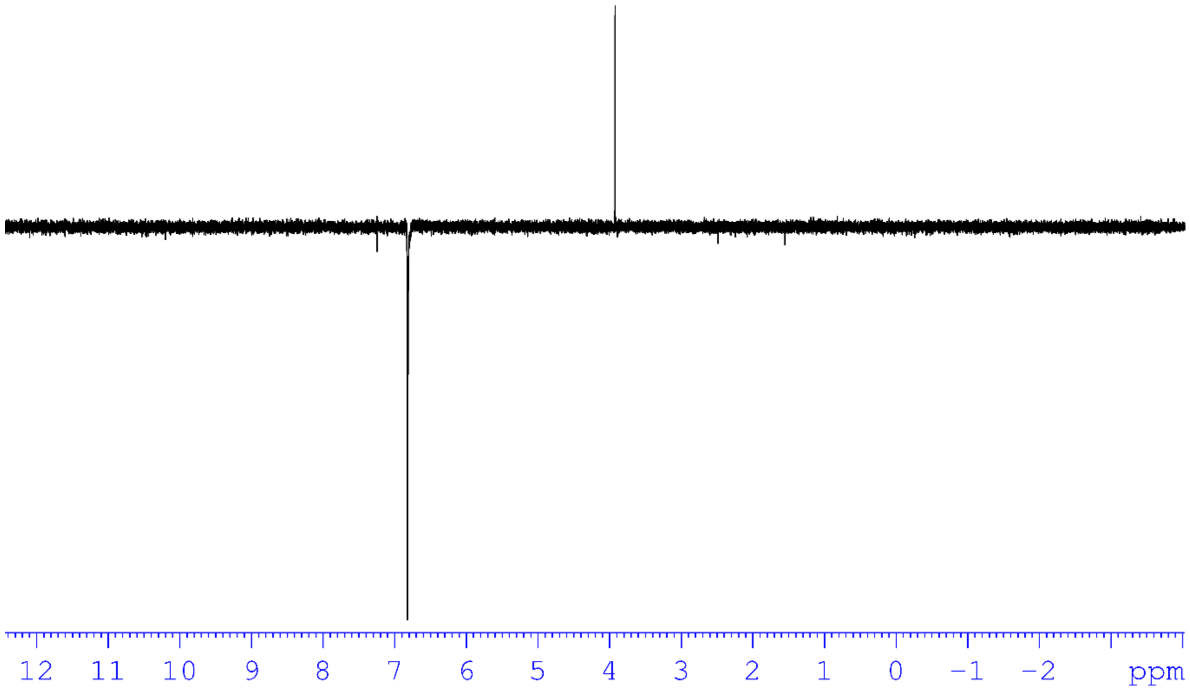

Figure S35. 1D selective NOESY (δ_H 6.84) spectrum of **5**.

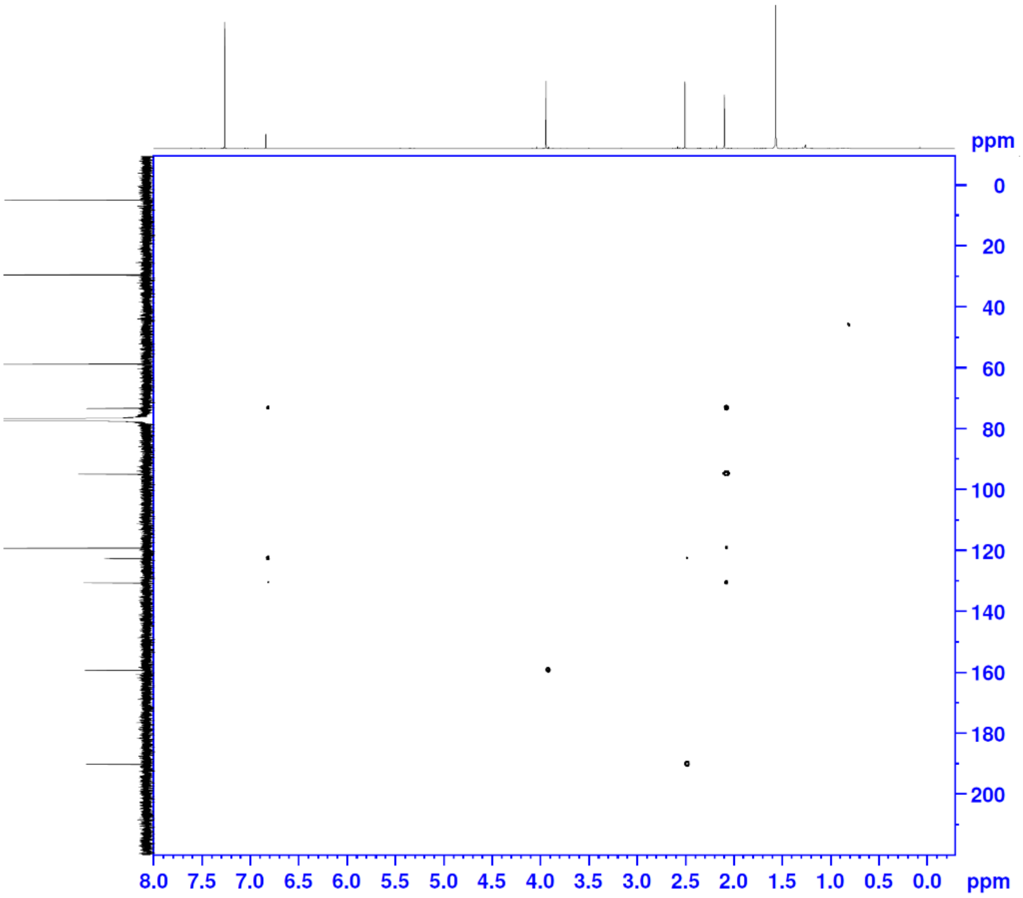

Figure S36. HMBC spectrum of **5**.

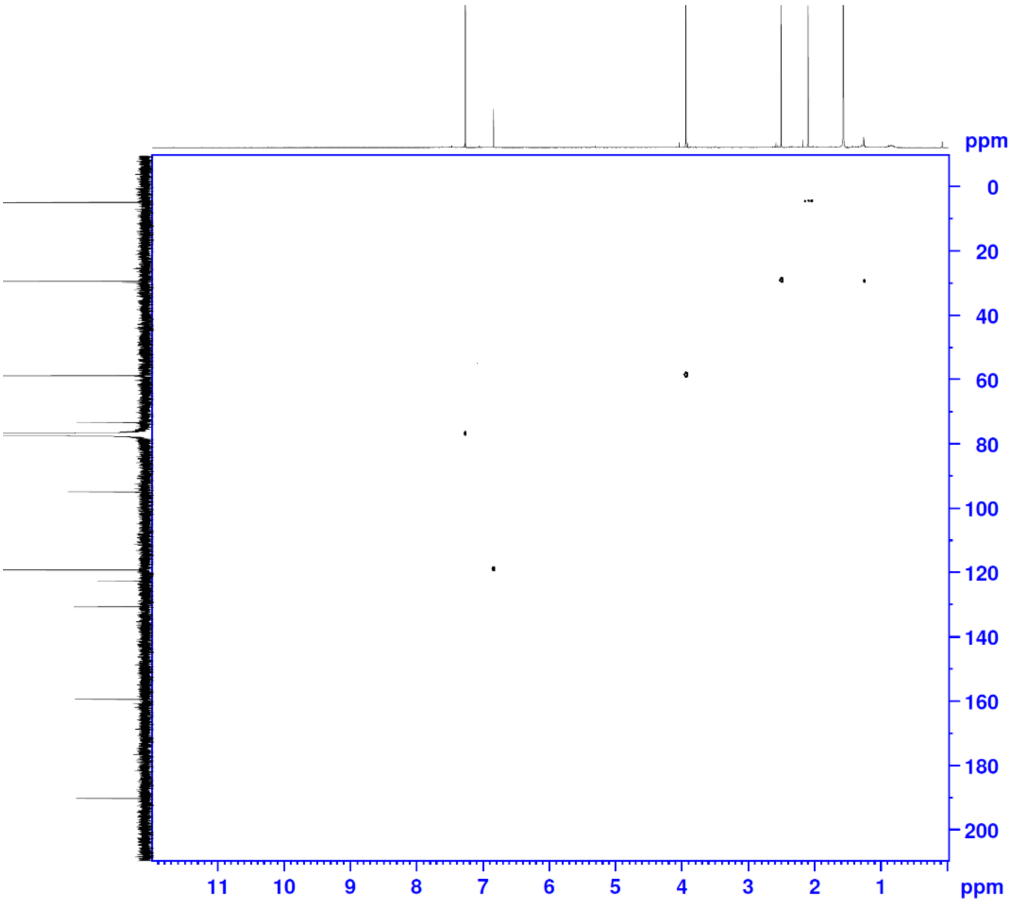

Figure S37. HSQC spectrum of **5**.
